# Beyond the textbook: Unveiling medical students’ voices on public health education

**DOI:** 10.4102/jcmsa.v3i1.180

**Published:** 2025-09-10

**Authors:** Tladi D. Ledibane, Marietjie de Villiers

**Affiliations:** 1 Department of Public Health Medicine, School of Medicine, Sefako Makgatho Health Sciences University, Garankuwa, South Africa; 2 Centre for Health Professions Education, Faculty of Medicine and Health Sciences, Stellenbosh University, Stellenbosch, South Africa

**Keywords:** medical education, public health curriculum, student perceptions, mixed-methods research, curriculum development

## Abstract

**Background:**

Training medical students in public health (PH) is crucial for shaping their future practices as healthcare professionals. This study aimed to explore the knowledge, attitudes and perceptions of fifth-year medical students at a South African university regarding their PH training to inform curriculum development and enhance the learning experience.

**Methods:**

A mixed-methods study with a convergent design was conducted. The questionnaire was distributed electronically to 248 fifth-year medical students using a 5-point Likert scale. A total of 85 students participated in the survey (34% response rate). Qualitative data were obtained through a single focus group discussion with five participants, all of whom were former class representatives from the same cohort and eligible survey participants. Descriptive statistics were used for quantitative data analysis, whereas thematic analysis was employed for qualitative data.

**Results:**

The study revealed appreciation for PH among students. However, concerns were raised about late exposure to PH in medical training and, predominantly, didactic teaching methods. Most students agreed on the integral role of PH in clinical medicine. The study also highlighted a need for more research opportunities in the undergraduate programme.

**Conclusions:**

The findings underscore the need for early exposure to PH, more engaging teaching methods and increased research training opportunities in PH. These insights can guide the revision and improvement of PH curricula in medical schools, potentially enhancing students’ preparedness for future roles in healthcare and research.

**Contribution:**

This study provides evidence from medical students’ perspectives to inform curriculum renewal in public health education, underscoring the need for earlier exposure, more engaging pedagogy, and integrated research training.

## Introduction

The coronavirus pandemic has thrust public health (PH) into the spotlight, with regular reports on epidemiological modelling, studies and disease outbreak control. The general public became more aware of PH practices and the role of PH professionals in the fight against the pandemic.^1^ The renewed interest has placed responsibility on PH academics to provide students with the necessary knowledge and skills to be effective clinicians who can play a meaningful role in the fight against the coronavirus disease 2019 (COVID-19) pandemic and future pandemics.^2^

Studies have shown that medical students understand and appreciate the value of PH in the undergraduate curriculum of medical schools.^3,4,5^ However, the students identified several factors that influenced their negative attitudes and perceptions of PH: poor educational experiences in PH training, a lack of positive role models, particularly exposure to PH medicine specialists, an overemphasis on statistics and epidemiology and negative attitudes towards PH topics.^2,5^

Students’ performance, satisfaction and success in medical school are related to their perceptions of the educational environment in which they learn.^6^ Experiential learning and innovative teaching approaches, such as community-based projects and group discussions, can improve the educational experience in the PH curriculum.^2,4^

Research on students’ knowledge, attitudes and perceptions about their PH training might provide insight and help provide evidence to inform and guide curriculum renewal. However, these curriculum changes should include students’ voices, and there is a need to evaluate students’ perceptions and views regarding their preferred learning environment before implementing changes to the curriculum.^2,6^

This study aimed to determine the knowledge of fifth-year medical students in PH after completing a PH training course, explore their attitudes and perceptions towards PH training and make recommendations for improvements.

## Research methods and design

### Research settings and context

The research was conducted in 2020 at a South African university (hereafter referred to as University X for ethical purposes). The 6-year undergraduate training of medical doctors consists of preclinical and clinical phases. The preclinical phase spans from the first year to the third year, and the clinical phase starts in the fourth year and ends in the sixth year. The students are introduced to PH and epidemiology fundamentals in the preclinical phase. Basic and applied PH principles are taught in the clinical phase, in the fourth year of study, through didactic whole-class lectures. There are no clinical rotations or experiential learning. The four modules are health measurement, health systems management, occupational medicine and the control of communicable and noncommunicable diseases. Students write four summative assessments at the end of each module and an examination at the end of the academic year.

The researchers used a convergent mixed-methods study design, which allows integrative analysis and interpretation of the results.^7^ The quantitative and qualitative phases were descriptive, cross-sectional and phenomenological, respectively. The target population for the quantitative phase included all 248 fifth-year medical students registered at University X in 2020. An electronic message was sent to the students, including a link to the informed consent documents and the survey questionnaire, which remained open for 8 weeks. For the qualitative phase, the target population consisted of eight former students and class representatives. Email invitations were sent to fifth-year medical students who had previously served as class representatives during their third and fourth years of study to participate in a focus group discussion (FGD).

The questionnaire was compiled based on a literature review to identify existing survey scales or items that could be used or modified.^8^ The questionnaire was reviewed by two PH medicine consultants and piloted with two PH medicine registrars before the students were emailed. The questionnaire explored demographics, knowledge, attitudes and perceptions regarding the PH training via a 5-point Likert scale. The researchers developed an interview guideline for the FGD.

The researchers conducted a single FGD, which was audio recorded, transcribed and anonymised prior to analysis.

Ethical clearance was obtained from Stellenbosch University and University X. Students signed informed consent before participation, and the data were anonymised.

Descriptive statistics were used to report quantitative data. The quantitative data were analysed via the Stata 12.00 (StataCorp., College Station, TX, USA) statistical software package. Summary statistics were prepared to describe continuous variables, and frequency tables were used to summarise categorical data.^2^

The qualitative data were analysed via thematic analysis methods.^9^ Firstly, the researchers became familiar with the data by repeatedly reading the transcriptions and listening to the focus group interview’s voice recording, as Sutton and Austin mentioned.^10^ Secondly, the researchers created initial codes for all the data and collated the data associated with each code.^10^ Thirdly, the researchers identified the themes and all related data. Fourthly, the themes associated with the coded extracts were established and expanded into a thematic map.^9^ Fifthly, the researchers defined the themes and assigned them names. Sixthly, a report summarising the findings of the analyses of the research question using relevant and important literature was produced.^2,9^

The researchers considered various perspectives throughout the thematic analysis process and incorporated reflections and notes into the report.

### Ethical considerations

The Stellenbosch University Health Research Ethics Committee (HREC) approved this study (S19/10/279). The Research Ethics Committee of University X ratified the study. University X requested that the study site be anonymised. The study adhered to the Declaration of Helsinki principles.^11^

For the quantitative phase of the investigation, an email was sent to all fifth-year medical students outlining the reasons for conducting the study. To access the questionnaire, the students were required to provide their electronic consent.

The researchers informed potential research participants about the nature and objective of the study, their rights regarding voluntary participation, and the methods used to ensure confidentiality and anonymity. Each participant completed and signed a consent form before the start of the focus group interview. The 2020 fifth-year cohort was selected for the study because it had recently completed its PH training in the preceding year and would not have any contact with researchers and the department for the remainder of their medical training.

#### Consent for publication

All participants provided consent for their anonymised data to be published.

## Results

Eighty-five (85) students out of a total of 248 responded to the online questionnaire, representing a response rate of 34%. The mean age of the participants was 24 years, ranging from 22 to 38 years. There were 61 (71.8%) female respondents and 24 (28.2%) male respondents. The focus group participants comprised three female students and two male students, aged from 22 to 25 years.

Table 1 shows the students’ attitudes towards the PH training. Most survey respondents (71 out of 85 (83.5%)) strongly agreed that the medical and PH fields collaborate to benefit patient care. Seventy participants (82%) agreed that doctors are an important part of the PH system, and 69 (81%) agreed that doctors play an important role in patient education. Only 41 (48%) survey participants strongly agreed that doctors should be required to learn PH.^2^

**TABLE 1:** General perceptions of students regarding public health.

General perceptions	Strongly agree		Agree		Do not know		Disagree		Strongly disagree	
	*n*	%	*n*	%	*n*	%	*n*	%	*n*	%
The medical and public health fields working together benefit patient care.	71	83.50	14	16.5	0	0.0	0	0.0	0	0.0
Doctors are part of the public health system.	70	82.00	14	16.0	0	0.0	1	1.0	0	0.0
Doctors play an important role in patient education.	69	81.10	15	17.7	0	0.0	1	1.2	0	0.0
Public health education is relevant to your career as a doctor.	63	74.10	22	25.9	0	0.0	0	0.0	0	0.0
Doctors play an important role in health education and disease prevention.	63	74.10	0	0.0	18	21.0	3	3.5	0	0.0
It is important for doctors to learn about population health strategies.	62	73.00	23	27.0	0	0.0	0	0.0	0	0.0
Public health education benefits clinical care.	60	70.60	23	27.1	0	0.0	2	2.6	0	0.0
Doctors should be required to learn public health.	41	48.20	38	44.7	2	2.6	4	4.7	0	0.0
Public health education in medical school changes and/or enforces policy.	32	37.70	35	41.2	17	20.0	0	0.0	1	1.2
Public health information is common-sense knowledge.	7	8.24	26	30.6	11	12.9	38	44.7	3	3.5

*Source*: Adapted from Ledibane TD. Knowledge, attitudes and perceptions of fifth-year medical students at a South African university regarding their public health course [MMed thesis] [homepage on the Internet]. Stellenbosch: Stellenbosch University; 2022 [cited 2025 Jul 31]. Available from: https://sun.primo.exlibrisgroup.com/discovery/fulldisplay?docid=alma999069485503436&context=L&vid=27US_INST:27US_V1&lang=en&search_scope=Combined&adaptor=Local%20Search%20Engine&tab=Everything&query=any,contains,knowledge%20attitudes%20and%20perception%20of%20medical%20students&offset=0

Table 2 shows the respondents’ views on important topics that should be included in the PH curriculum. All survey respondents agreed or strongly agreed that maternal and child health, epidemiology, communicable diseases and health promotion are important PH topics that should be covered in the PH curriculum.^2^

**TABLE 2:** Perceptions about public health topics relevant to the medical curriculum (*N* = 85).

Public health topics	Strongly agree		Agree		Don’t know		Disagree		Strongly disagree	
	*n*	%	*n*	%	*n*	%	*n*	%	*n*	%
Mother and child health	69	81.2	16	18.2	0	0.0	0	0.0	0	0
Communicable diseases	66	77.7	19	22.4	0	0.0	0	0.0	0	0
Health promotion	62	72.9	23	27.1	0	0.0	0	0.0	0	0
Noncommunicable diseases	52	61.2	30	35.3	3	3.3	0	0.0	0	0
Public health policy	40	47.1	38	44.7	6	7.1	1	1.2	0	0
Epidemiology	35	41.2	43	50.6	6	7.1	1	1.2	0	0
Occupational medicine	35	41.2	41	48.2	7	8.2	2	2.6	0	0
Health services administration	34	40.0	34	40.0	14	14.0	3	3.6	0	0
Cultural competency and diversity	34	40.0	35	41.2	11	12.9	5	5.9	0	0
Population health	27	31.7	44	51.8	10	11.8	4	4.7	0	0

*Source*: Adapted from Ledibane TD. Knowledge, attitudes and perceptions of fifth-year medical students at a South African university regarding their public health course [MMed thesis] [homepage on the Internet]. Stellenbosch: Stellenbosch University; 2022 [cited 2025 Jul 31]. Available from: https://sun.primo.exlibrisgroup.com/discovery/fulldisplay?docid=alma999069485503436&context=L&vid=27US_INST:27US_V1&lang=en&search_scope=Combined&adaptor=Local%20Search%20Engine&tab=Everything&query=any,contains,knowledge%20attitudes%20and%20perception%20of%20medical%20students&offset=0

Table 3 displays respondents’ perspectives on teaching methods that should be included in the PH training. Hands-on projects in PH and tutorials should be used to teach PH, according to 42 (49.4%) and 31 (36.5%) survey respondents, respectively. Twenty-five (29.4%) respondents agreed that the department should use online and blended learning in the PH training, whereas 23 (27.1%) agreed that the department should use formal face-to-face lectures. Fifty-four (51.8%) survey participants were unsure whether flipped classroom techniques should be used to teach PH.^2^

**TABLE 3:** Respondents’ perceptions regarding teaching public health.

Teaching methods	Strongly agree		Agree		Don’t know		Disagree		Strongly disagree	
	*n*	%	*n*	%	*n*	%	*n*	%	*n*	%
Hands-on projects in public health	42	49.4	34	40.0	6	7.1	3	3.50	0	0.0
Tutorial method	31	36.5	42	49.4	6	7.1	4	4.71	2	2.4
Assignments	28	28.0	35	35.0	5	5.9	16	18.80	1	1.2
Online teaching	25	29.4	35	41.2	6	7.1	17	20.00	2	2.4
Blended learning	25	29.4	44	51.8	12	14.1	4	4.71	0	0.0
Formal face-to-face lectures	23	27.1	37	43.5	6	7.1	16	18.90	3	3.5
Self-study method	12	14.1	20	23.5	11	12.9	30	35.30	12	14.1
Flipped classroom*	5	5.9	23	27.1	44	51.8	11	12.90	2	2.4

*Source*: Adapted from Ledibane TD. Knowledge, attitudes and perceptions of fifth-year medical students at a South African university regarding their public health course [MMed thesis] [homepage on the Internet]. Stellenbosch: Stellenbosch University; 2022 [cited 2025 Jul 31]. Available from: https://sun.primo.exlibrisgroup.com/discovery/fulldisplay?docid=alma999069485503436&context=L&vid=27US_INST:27US_V1&lang=en&search_scope=Combined&adaptor=Local%20Search%20Engine&tab=Everything&query=any,contains,knowledge%20attitudes%20and%20perception%20of%20medical%20students&offset=0

* Instead of having a lecture on how to calculate disease incidence, you’d watch a video explaining the concept prior to attending class.

The qualitative data further informed the survey findings. Using the thematic analysis approach, the researchers identified three emerging themes, namely the ‘scope of public health practice’, ‘the teaching and learning experience’ and ‘attitudes towards their public health training’.

The following particularly expressive and representative quotes by the participants are listed as exemplary. The quotes illustrate the different perceptions regarding the training. Among the eight prospective previous class representatives, five consented to participate in the study.

### The scope of public health practice

#### Population-level interventions

The focus group participants had similar perspectives regarding the scope of PH practice, that is, PH targets the population or communities regarding its interventions. Participants P02 and P01 stated that:

‘Public health deals with diseases or disorders that commonly affect the community at large instead of disorders that are just specific to just an individual.’ (P02)

‘[*M*]y understanding of public health discipline is that it is a discipline in medicine which deals with eh! population, the health of a population rather than an individual.’ (P01)

#### Prevention of disease

The focus group participants also presented perspectives on the disease prevention role of PH at the community level as opposed to the curative care aimed at individuals.

‘It is like community-based where it deals with prevention at a level of population that we do not have to burden the health system with “curing” [*curative care*].’ (P01)

Another participant explained:

‘Let me give you a practical example with … cholera; because of public health and education and prevention, now people even who drink water from the river or from the streams they know that they must put jik [*a detergent*] in it first, for I think about seven days, or they must boil it first to prevent cholera that will also reduce the deaths because of that disease.’ (P02)

### The teaching and learning experience

#### The timing of exposure to public health

One student expressed the view that the PH training should be introduced in the first year of study to align with the community outreach training, which involves conducting house visits and delivering health talks to families and community members. The participant advocated for early exposure to PH to help prepare and integrate with their community outreach block:

‘[*Y*]ou come from high school where you were doing Biology, Maths and English and what … and you are expected to give a health talk on HIV, a health talk on TB, a health talk on you know … they ask you to choose a topic but, you have not had a lecture on anything because what you are doing in first year is Physics, Chemistry and all the nonclinical things.’ (P01)

‘Eh! For me honestly, because I also like public health, I found it very important, but the only problem I have is the fact that we are doing it only in the fourth year.’ (P01)

#### Integration with other modules

The focus group participants P04 and P01 felt that early exposure to PH would foster better integration with the Medical Humanities (MEHS) block. (Medical Humanities is a module in which students attend primary healthcare clinics, conduct house visits, offer health talks and learn basic clinical skills.)

‘And then another thing in first year there is a block ja [of] MEHS, where we go out into the community neh! So a course like this will actually be beneficial because one of the requirements of the MEHS block is to give a health talk to the community so that this could play a very big role in that.’ (P04)

‘In such cases where we could do Community Health and learn about these conditions that affect the public, then you will even do better health talks with better understanding also.’ (P01)

#### Research training

The focus group participants felt that they were not receiving adequate training and exposure in research, and that research training should be included in the PH training.

The focus group participants P04 and P02 felt that there were insufficient research opportunities for undergraduates at University X.

‘I don’t feel like we are truly, truly prepared enough for research. So, it is something that if it can be incorporated in public health, I think it will be very useful.’ (P04)

‘And I also think that we as new graduates; we are not adequately prepared for research.’ (P02)

#### Learning opportunities

The participants expressed concern about teaching strategies that were not engaging or interactive because some lecturers simply read slides to them and lacked experiential learning.

‘You know you are gonna attend, and somebody is like maybe reading the slides about Community Health neh! If you can incorporate like a group discussion where maybe you can give them an assignment to say, okay, if you guys were to go now to the community and to tell them about HIV, how would you present it? wa bona and go na le [you see and you have] like a small presentation and you can do it like groups come and present a condition or something. Uhm! That would make it more interactive, and you can actually remember from those presentations and good presentations or discussions.’ (P02)

Another participant added:

‘I do not think it [public health training] is enough for an undergraduate who is coming into university for the first time …like I say it is not even a practical component to just read on the units that are provided, and then the fact that it is just like you reading theory, that does not do much for a person.’ (P01)

Another participant added:

‘We want contact classes in addition to online classes and practical exposure too in public health.’ (P05)

### Attitudes towards their public health training at University X

#### Competing academic pressures

Considering the workload in MBChB IV, one participant felt that the students needed to focus on ‘hard’ courses and could read up on PH and still do well in assessments:

‘I can just read because it is not as difficult to read public health on your own, as much as it is important, but I can read about cholera and still answer a few questions in the exams. So, students will rather go and concentrate on a difficult course than, not that they are not the same, but I am failing pharmacology; I would rather concentrate on that.’ (P01)

## Discussion

The results of this study shed light on the perceptions and attitudes of medical students towards their PH training. The findings reveal that most of the respondents appreciate the importance of PH in their medical training. However, the timing of exposure to PH and the teaching methods used in delivering the training content were areas of concern for the students.

The late introduction of PH training in the medical curriculum was a concern for most students. They believed that early exposure to PH concepts would allow for better integration with other medical courses and provide a comprehensive understanding of its relevance in clinical practice.^12^ However, this finding is in contrast with the findings by Zweigenthal and colleagues, who reported that postgraduate students’ appreciation of PH was often shaped by later clinical experiences, not early undergraduate exposure.^13^ On the other hand, the advent of the COVID-19 pandemic may have led to the students appreciating the role of PH in clinical practice early on and thus shaped their favourable views towards PH,^1^ even though PH critical content remains poorly integrated or introduced late into the curriculum.^14^

Early introduction of PH aligns with the competencies outlined by the Health Professions Council of South Africa (HPCSA), which are derived from the Canadian Medical Education Directives for Specialists (CanMEDS) framework.^15^ These include the roles of medical expert, health advocate and scholar, each of which requires foundational knowledge in PH to address population-level health needs. Furthermore, early integration should be longitudinal across all the years of medical training lest students view PH as less legitimate than ‘hard’ clinical courses and thus fail to prioritise it when dealing with competing academic pressures.

The teaching methods used in delivering the PH content were another area of concern for the students. The students expressed the need for more engaging and interactive teaching strategies. Specifically, the FGD participants advocated the use of pedagogical strategies such as group learning and integrated case studies. These findings align with the literature that suggests the use of active learning strategies to enhance student engagement and understanding of PH concepts.^16,17^

Despite these concerns, most students agreed that PH is an integral part of clinical medicine that should be included in undergraduate medical training. This finding was consistent with the opinions of several PH authors^18,19^ and the findings of studies conducted elsewhere.^3,20^ This positive perception of PH is encouraging and supports ongoing efforts to strengthen PH training in medical education.^21^

Moreover, the current findings point to a need for the integration of research skills training within the undergraduate PH curriculum. Students expressed concern about being ill-prepared for post-graduation research. Prior work by Adefolalu and colleagues similarly found that students lack sufficient exposure and support to pursue research during medical school.^22^ Structured research exposure, embedded in PH teaching, could help address this concern.

The findings of this study support the widely held perception among medical students that PH is an easy and less demanding subject when compared to other clinical courses.^4,5^ This perception may result in students allocating less time and effort to PH in favour of subjects they view as more challenging. However, strategies such as integrating PH throughout the curriculum and adopting assessment practices such as authentic assessments (tasks that mimic real-life problems)^23^ competency-based assessments and formative assessments with feedback can enhance student learning and promote better student engagement with the course content.^24,25^

### Limitations of the study

This study employed a convergent mixed-methods design, with both quantitative and qualitative data collected from the same student cohort. A total of 248 fifth-year students were invited to participate in the online survey, and 85 students responded (response rate = 34%).^2^ Additionally, a single FGD was conducted with five participants, all of whom were former class representatives from the same cohort and were not excluded from the survey phase.

The overlap between the FGD and survey participants, while methodologically coherent, presents a limitation in terms of representativeness. These students may have been more engaged or interested in PH education, thereby introducing a positive response bias. The low survey response rate further limits generalisability, both within the studied institution and to other South African medical schools. Furthermore, selection bias may have resulted in overrepresentation of students with favourable views towards PH, while dissenting or indifferent voices may be underrepresented.

## Conclusion

This study provides potentially valuable insights into the perceptions and attitudes of medical students towards their PH training. The findings highlight the need for early but integrated exposure to PH across the curriculum, interactive and experiential teaching strategies and the inclusion of structured research training.
